# Head Lice of Pygmies Reveal the Presence of Relapsing Fever Borreliae in the Republic of Congo

**DOI:** 10.1371/journal.pntd.0005142

**Published:** 2016-12-02

**Authors:** Nadia Amanzougaghene, Jean Akiana, Géor Mongo Ndombe, Bernard Davoust, Nardiouf Sjelin Nsana, Henri-Joseph Parra, Florence Fenollar, Didier Raoult, Oleg Mediannikov

**Affiliations:** 1 Unité de Recherche sur les Maladies Infectieuses Tropicales Emergentes (URMITE), Aix-Marseille Université, Marseille, France; 2 Laboratoire national de santé publique, Brazzaville, République du Congo; 3 Campus International UCAD-IRD, Dakar, Senegal; Baylor College of Medicine, UNITED STATES

## Abstract

**Background:**

Head lice, *Pediculus humanus capitis*, occur in four divergent mitochondrial clades (A, B, C and D), each having particular geographical distributions. Recent studies suggest that head lice, as is the case of body lice, can act as a vector for louse-borne diseases. Therefore, understanding the genetic diversity of lice worldwide is of critical importance to our understanding of the risk of louse-borne diseases.

**Methodology/Principal Findings:**

Here, we report the results of the first molecular screening of pygmies’ head lice in the Republic of Congo for seven pathogens and an analysis of lice mitochondrial clades. We developed two duplex clade-specific real-time PCRs and identified three major mitochondrial clades: A, C, and D indicating high diversity among the head lice studied. We identified the presence of a dangerous human pathogen, *Borrelia recurrentis*, the causative agent of relapsing fever, in ten clade A head lice, which was not reported in the Republic of Congo, and *B*. *theileri* in one head louse. The results also show widespread infection among head lice with several species of Acinetobacter. *A*. *junii* was the most prevalent, followed by *A*. *ursingii*, *A*. *baumannii*, *A*. *johnsonii*, *A*. *schindleri*, *A*. *lwoffii*, *A*. *nosocomialis* and *A*. *towneri*.

**Conclusions/Significance:**

Our study is the first to show the presence of *B*. *recurrentis* in African pygmies’ head lice in the Republic of Congo. This study is also the first to report the presence of DNAs of *B*. *theileri* and several species of Acinetobacter in human head lice. Further studies are needed to determine whether the head lice can transmit these pathogenic bacteria from person to another.

## Author Summary

Head lice, *Pediculus capitis humanus*, and body lice, *Pediculus h*. *humanus*, are obligatory ectoparasites that feed exclusively on human blood. Currently, the body louse is the only recognized vector of at least three deadly bacterial pathogens that have killed millions of peoples, namely: *Rickettsia prowazekii*, *Bartonella quintana* and *Borrelia recurrentis*, responsible for epidemic typhus, trench fever and relapsing fever, respectively. In this work, we aimed to study the genetic diversity of head lice collected from African Pygmies in the Republic of Congo and to look for louse-borne pathogens in these lice. We detected *B*. *recurrentis* in head lice belonged to clade A that is prevalent in the Republic of Congo. Our study also show, for the first time, the presence of DNAs of *B*. *theileri* and several species of *Acinetobacter* in human head lice. Despite several investigations into the transmissibility of numerous infectious agents, no conclusive evidence has demonstrated the transmission of disease by head lice. That said, we believe that pathogens detected in head lice may be an indirect tool for evaluating the risk of louse-borne diseases in humans.

## Introduction

The head louse, *Pediculus humanus capitis*, and the body louse, *P*. *h*. *humanus*, are obligatory hematophagous parasite that thrived exclusively on human blood for thousands of years [1, 2]. The two lice are now usually considered members of a single species as opposed to separate species [3, 4], each louse lives and multiplies in a specific ecological niche: hair for head lice and clothing for body lice [5, 6].

Molecular analysis of mitochondrial genes has permitted the classification of *Pediculus humanus* into three several clades or haplogroups, referred to as A, B, and C [1, 2, 7, 8, 9]. Haplogroup A is the most common, and possesses a global distribution, including both head and body lice [1, 2, 6, 8, 9]. Clade B comprises only head lice, is confined to the New Word, Europe, Australia and was recently reported in North and South Africa [2, 6, 10, 11]. Clade C includes only head lice and is mainly found in Africa and Asia [2, 5, 9, 10]. Most recently, a novel clade D, comprising both head and body lice, was described in Democratic Republic of the Congo [6].

Prior research suggested that the known lice clades evolved on different lineages of *Homo*, similarly to those which are known to have existed 2.3 to 0.03 million years ago (MYA) [1, 11], and accordingly their geographic distribution may provide information regarding the evolutionary history of the lice as well as their human hosts [1, 2, 22]. Clade A lice are most likely to have emerged in Africa and to have evolved on the host linage that led to anatomically modern humans (*Homo sapiens*), showing the signs of a recent demographic expansion out of Africa about 100,000 years ago, first to Eurasia and subsequently to Europe, Asia, and the New World [1, 5, 12]. Haplogroup B diverged from haplogroup A between 0.7 and 1.2 MYA and may have evolved on archaic hominids, such as the *Homo sapiens neanderthalensis*, who spread across Europe and Asia, only becoming associated with modern humans during the period of overlap as the result of a recent host switch [1, 5, 12].

Head lice are one of the most prevalent parasitic infestations in contemporary populations, particularly in children. They often cause intense itching and, in some cases, insomnia. As a result, they represent a major economic and social concern worldwide [6, 13, 14]. Body lice, unlike head lice, are nowadays less prevalent and tend to appear mainly in indigent individuals living in poor sanitary conditions [6, 9, 13]. They do, however, present a far more serious threat to public health because they transmit at least three deadly bacterial pathogens that have killed millions of peoples, namely: *Rickettsia prowazekii*, *Bartonella quintana*, and *Borrelia recurrentis*, responsible for epidemic typhus, trench fever, and relapsing fever, respectively [5, 9, 13]. Body lice are also suspected of transmitting the agent of plague, *Yersinia pestis* and the nosocomial pathogen, *Acinetobacter baumannii* [6, 15, 16].

Until recently, it was believed that head lice cannot transmit louse-borne diseases [17]. Recently, however, its status as a vector of pathogens has been brought into question, since, they have been found to carry the DNA of *B*. *quintana*, *B*. *recurrentis*, *A*. *baumannii*, and *Y*. *pestis* in natural settings [6, 18, 19, 20, 21, 22, 23]. Furthermore, experimental infections have shown that head lice may also act as a vector of louse-borne diseases [24, 25], justifying a detailed understanding of their genetic diversity and distribution worldwide.

In Central Africa, studies on head lice, particularly those involving indigenous individuals, have received little prior attention. Of these indigenous populations, the African Pygmies are hunter-gatherers who live scattered in the equatorial forest. They are characterized by having a very short stature [26]. The Eastern and Western Pygmies represent the two principal groups of African Pygmies [26]. The Western group is estimated to include 55,000 individuals living in the Western Congo basin, across the countries of Cameroon, Republic of Congo, Gabon and Central African Republic, and its subgroups are identified by different names, including the Binga, Baka, Biaka and Aka or Atsua [26].

Furthermore, the detection of *B*. *recurrentis* in African lice remains limited to only a small number of countries. Currently, this bacterium is endemic in Eastern Africa (Ethiopia, Eritrea, Somalia, and Sudan) with the highest number of cases observed in Ethiopia, where it is the seventh most common cause of hospital admission and the fifth most common cause of death [27, 28]. Nevertheless, this borreliae has not been reported in any of the Central African countries cited above.

In this work, we aimed to study the genetic diversity of head lice collected from African Pygmies in the Republic of Congo and to look for louse-borne pathogens in these lice.

## Materials and Methods

### Ethics statement and louse sampling

This study was approved by the Health Ministry of the Republic of Congo (000208/MSP/CAB.15 du Ministère de la Santé et de la Population, 20 August 2015). All necessary permits were obtained from the individuals involved or their legal representatives in the case of children. All permissions were granted orally, because the participants are illiterate. The representatives of a local Health Center and the village elders accompanied the researchers to ensure that information was correctly translated into local languages and that the villagers were willing to take part in the study.

**Fig 1:**
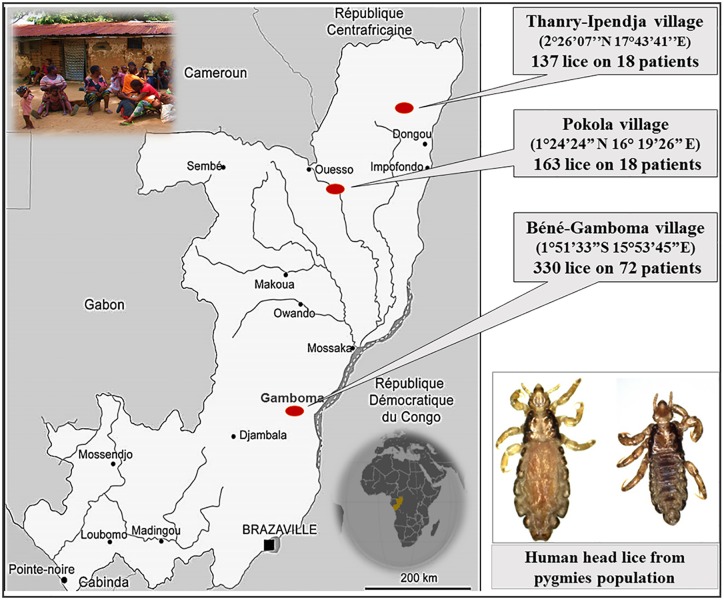
Map of head lice collection in the pygmy population from Congo-Brazzaville.

A total of 630 head lice samples were collected from 126 apparently healthy authochthonal individuals (pygmies) in the Republic of Congo (Congo-Brazzaville) in August 2015. The collections were conducted in three different villages: i) Thanry-Ipendja, where 137 lice were isolated from 18 people, ii) Pokola, where 163 lice were isolated from 36 people, and iii) Béné-Gamboma, where 330 lice were isolated from 72 people (Fig 1). All the sampled individuals were thoroughly examined for the presence of both head and body lice. All visible head lice were removed from hair using a fine-tooth comb. Lice were then collected from the clean white tissue with forceps. No body lice were found during the examination. All the lice were preserved in 70% ethanol and transported to our laboratory in Marseille (France).

### DNA extraction

The head lice specimens were removed from the 70% ethanol, washed three times in distilled water, and cut in half. The genomic DNA of each half louse was extracted using a DNA extraction kit, QIAamp Tissue Kit (Qiagen SAS, Courtaboeuf, France) with the EZ1 apparatus following the manufacturer’s protocols. The extracted head lice DNA was assessed for quantity and quality using a Nano Drop spectrophotometer (Thermo Scientific, Wilmington, United Kingdom). The genomic DNA was stored at -20°C under sterile conditions until the next stage of the investigation.

### Genotypic status of lice

#### Determination of louse clade by real-time PCR assays

In order to identify the clades of the collected lice, we developed a real-time quantitative PCR (qPCR) method based on two duplex designed from the cytochrome *b* (*cytb)* gene. The first duplex consisted of a set of primers with FAM-and VIC- labeled probes specific to clade A and D respectively, targeting 140-bp of *cytb* (nucleotide position 190–329 of *cytb* gene). The second duplex consisted of another set of primers with FAM-and VIC- labeled probes specific to clade B and C, respectively, targeting 187-bp of the *cytb* gene (nucleotide position 499–685 of *cytb* gene). All available sequences of *cytb* of the four mitochondrial clades of human lice were aligned by CLUSTAL X 2.0.11 [29] and signature sites of each clade were identified. The following design was based on identified signature sites and performed with Primer3 software, version 4.0 (http://frodo.wi.mit.edu/primer3/), following the general rules described elsewhere [30]. Sequences of primers and probes are shown in Table 1.

**Table 1:** Oligonucleotide sequences of primers and probes used for real-time PCRs and conventional PCRs in this study.

Target	Name	Primers (5’-3’) and probes	Source
***Pediculus humanus***			
cytochrome b	Duplex A-D	F_ GATGTAAATAGAGGGTGGTT	This study
		R_ GAAATTCCTGAAAATCAAAC	
		FAM-CATTCTTGTCTACGTTCATATTTGG-TAMRA	
		VIC-TATTCTTGTCTACGTTCATGTTTGA-TAMRA	
	Duplex B-C	F_ TTAGAGCGMTTRTTTACCC	This study
		R_ AYAAACACACAAAAMCTCCT	
		FAM-GAGCTGGATAGTGATAAGGTTTAT-MGB	
		VIC-CTTGCCGTTTATTTTGTTGGGGTTT-TAMRA	
	*Cytb*	F_GAGCGACTGTAATTACTAATC	[31]
		R_CAACAAAATTATCCGGGTCC	
***Rickettsia* spp.**			
citrate synthase (*gltA*)	RKNDO3	F_GTGAATGAAAGATTACACTATTTAT	[32]
		R_GTATCTTAGCAATCATTCTAATAGC	
		FAM-CTATTATGCTTGCGGCTGTCGGTTC-TAMRA	
***Acinetobacter spp*.**			
RNA polymerase β subunit gene	*rpoB*	F_TACTCATATACCGAAAAGAAACGG	[18]
		R_GGYTTACCAAGRCTATACTCAAC	
		FAM-CGCGAAGATATCGGTCTSCAAGC-TAMRA	
	*rpoB* (zone1)	F_TAYCGYAAAGAYTTGAAAGAAG	[33]
		R_CMACACCYTTGTTMCCRTGA	
***Rickettsia prowazekii***			
*rOmpB* gene	*ompB*	F_AATGCTCTTGCAGCTGGTTCT	[34]
		R_TCGAGTGCTAATATTTTTGAAGCA	
		FAM-CGGTGGTGTTAATGCTGCGTTACAACA-TAMRA	
***Yersinia pestis***			
	PLA	F_ATG GAG CTT ATA CCG GAA AC	[34]
		R_GCG ATA CTG GCC TGC AAG	
		FAM-TCCCGAAAGGAGTGCGGGTAATAGG-TAMRA	
***Borrelia* spp.**			
*16S ribosomal RNA*	Bor16S	F_AGCCTTTAAAGCTTCGCTTGTAG	[35]
		R_GCCTCCCGTAGGAGTCTGG	
		FAM-CCGGCCTGAGAGGGTGAACGG-TAMRA	
flagellin gene	*flab*	F_GCTGAAGAGCTTGGAATGCAACC	[36]
		R_TGATCAGTTATCATTCTAATAGCA	
***Bartonella* spp.**			
Internal transcribed spacer 16S-23S	BartoITS2	F_GATGCCGGGGAAGGTTTTC	[18]
		R_GCCTGGGAGGACTTGAACCT	
		FAM-GCGCGCGCTTGATAAGCGTG-TAMRA	
***Anaplasma* spp.**			
23S ribosomal RNA	TtAna	F_TGACAGCGTACCTTTTGCAT	[37]
		R_TGGAGGACCGAACCTGTTAC	
		FAM-GGATTAGACCCGAAACCAAG-TAMRA	

In order to confirm the specificity of the qPCRs which were developed, both duplex qPCR assays were optimized and screened for specificity and sensitivity by testing louse specimens from known clades obtained from the private frozen collection of world lice belonging to our laboratory (URMITE). All of the 630 pygmy head lice specimens were then tested in both duplex qPCR assays.

The final reaction volume of 20 μl contained 5 μL of the DNA template, 10 μl of Eurogentec Probe PCR Master Mix (Eurogentec, Liège, Belgium), 0.5 mM of each primer and 0.5 mM of the FAM- and VIC labeled probes for each duplex. PCR amplification was carried out in a CFX96 Real-Time system (Bio-Rad Laboratories, Foster City, CA, USA) using the following thermal profile: one incubation step at 50°C for two minutes and an initial denaturation step at 95°C for three minutes, followed by 40 cycles of denaturation at 95◦C for 15 seconds and annealing extension at 60°C for 30 seconds. As positive controls, we used lice with known clades.

#### Cytochrome b amplification and sequencing

For phylogenetic study, DNA samples of approximately 20% of the total number of lice collected in each village were randomly selected to ensure an equal distribution of the included lice from the three villages studied. They were subjected to standard PCR targeting a 347-bp fragment of *cytb* gene as previously described [31].

PCR amplification was performed in a Peltier PTC-200 model thermal cycler (MJ Research Inc., Watertown, MA, USA). PCR reactions contained 5 μl of DNA template, 2.5 μl of Tampon Buffer, 1 μl of MgCl_2_, 2.5 μl 2 μM of dNTP, 0.5 μl 10 μM of each primer, 0.25 μl Hotstar Taq-polymerase (Qiagen) and water to create a final reaction mixture volume of 25 μl. The thermal cycling conditions were one incubation step at 95°C for 15 minutes, 40 cycles of one minute at 95°C, 30 seconds at 56°C and one minute at 72°C followed by a final extension for five minutes at 72°C. Negative and positive controls were included in each assay. The success of amplification was confirmed by electrophoresis on a 1.5% agarose gel. Purification of PCR products was performed using NucleoFast 96 PCR plates (Macherey-Nagel EURL, Hoerdt, France) as per the manufacturer’s instructions. The amplicons were sequenced using the Big Dye Terminator Cycle Sequencing Kit (Perkin Elmer Applied Biosystems, Foster City, CA) with an ABI automated sequencer (Applied Biosystems). The electropherograms which were obtained were assembled and edited using ChromasPro software (ChromasPro 1.7, Technelysium Pty Ltd., Tewantin, Australia) and compared with those available in GenBank database by NCBI BLAST (http://blast.ncbi.nlm.nih.gov/Blast.cgi).

### Molecular screening for the presence of pathogen DNA

The qPCR was performed to screen all lice samples using previously reported primers and probes for *Borrelia* spp., *Bartonella* spp., *Acinetobacter* spp., *Rickettsia* spp., *Rickettsia prowazekii*, *Y*. *pestis*, and *Anaplasma* spp. (Table 1). All qPCRs were performed using a CFX96 Real-Time system (Bio-Rad Laboratories) and the Eurogentec Master Mix Probe PCR kit (Eurogentec). We included the DNA of the target bacteria as positive controls and master mixtures as a negative control for each test. We considered samples to be positive when the cycle’s threshold (Ct) was lower than 35 Ct [38].

To identify the species of bacteria, all positive samples from qPCRs for *Acinetobacter* spp. and *Borrelia* spp. were further subjected to standard PCR, targeting a portion of the *rpoB* gene (zone1) and a portion of the *flab* gene, respectively, using the primers and all conditions as described previously [33, 36]. Successful amplification was confirmed via gel electrophoresis and amplicons were prepared and sequenced using similar methods as described for *cytb* gene for lice above.

### Data analysis

For comparison, the head lice DNA sequences obtained in this study were combined with the 30 *cytb* haplotypes reported by Drali *et al*. [39]. We then complemented this dataset with newly available sequences in GenBank, then assigned them to haplotypes using DnaSP v5.10 [40]. Finally, we created a dataset that consisted of 51 haplotypes. These haplotypes span 41 geographic locations (countries) in five continents (S1 Table).

In order to investigate the possible relationships between the haplotypes, the median-joining (MJ) network using the method of Bandelt was constructed with the program NETWORK4.6 (www.fluxus-engineering.com/sharenet.htm) [41].

Phylogenetic analyses and tree reconstruction were performed using MEGA software version 6.06 [42] with 500 bootstrap replications.

## Results

### Genetic status of lice

#### Identification of the specificity of two developed duplex qPCRs for the determination of lice clades

Two developed duplex qPCRs (A + D and B + C clades) were tested on 249 lice from the URMITE collection of those lice whose mitochondrial clades had already been identified by sequencing the portion of *cytb* gene [31]. In total, 249/249 lice produced fluorescence curves in qPCR. The clades were correctly identified in 249/249 cases.

#### Determination of lice clade by two duplex qPCRs

In total, 630 head lice were collected from 126 individuals living in three villages from different prefectures of Congo-Brazzaville, and all were tested by both the duplex q-PCRs to determine their clade. Our result showed that 431 (68.4%) lice belonged to clade A, 134 (21.3%) lice to clade C and only 65 (10.3%) lice to clade D. Considering the geographical regions where the lice were collected, all those collected from the villages of Pokola and Thanry-Ipendja belonged to clade A, while those collected from the village of Béné-Gamboma belonged to all three clades (Clade A, C, and D).

Of the 126 persons, 90 (71.41%) were mono-infested by only one clade of lice. Of these, 67 (53.17%) were only infested with lice from clade A, four (3.17%) were only infested with lice from Clade D, and 19 (15.07%) were exclusively infested with lice from Clade C. Dual infestation was observed in 23 individuals (18.25%), of which eight featured both Clade A and D, seven featured both Clade A and C, and eight featured both Clade D and C. Triple infestation for all three clades was detected in 13 people (10.31%) (Table 2).

**Table 2:** Number of pygmy individuals infested with single or multiple clades of lice from Congo-Brazzaville.

Clade of lice	Individual infested (n = 126)	
	no.	%
***Single infestation***		
Clade A	67	53.17
Clade D	4	3.17
Clade C	19	15.07
***Total***	**90**	**71.41**
***Multiple infestation***		
Clade A/D	8	6.35
Clade D/C	8	6.35
Clade C/A	7	5.55
Clade A/D/C	13	10.31
***Total***	**36**	**28.56**

### Phylogenetic analysis and haplotype assignment

**Fig 2:**
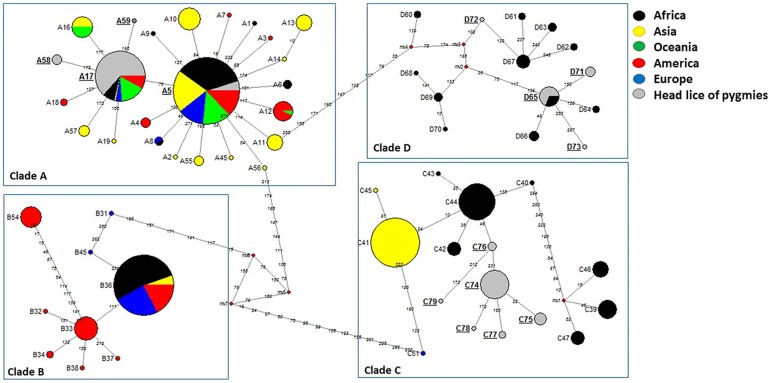
*Cytb* haplotype networks of human body and head lice. Each circle indicates a unique haplotype and variations in circle size are proportional to haplotype frequencies. Pie colors and sizes in circles represent the continents and the number of their sequence for a haplotype. The length of the links between nodes is proportional to mutational differences. Haplotypes identified in the present study are in bold.

**Fig 3:**
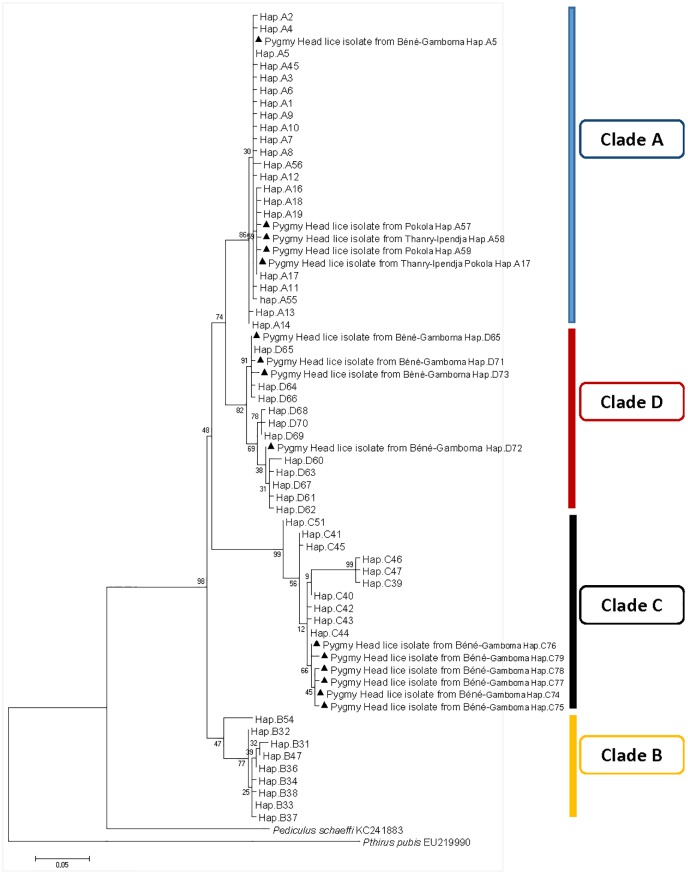
Maximum-likelihood phylogram of *Pediculus humanus* haplotypes based on partial 272-bp *cytb* gene with Pediculus schaeffi and Pthirus pubis as outgroups.

A total of 160 head lice *cytb* sequences were analyzed in this work yielding 83 variable positions defining 15 different haplotypes, including 11 new ones: five from haplogroup A (35.7%), four from haplogroup D (28.5%), and six from haplogroup C (42.8%) (Table 3). These haplotypes, together with references from all the body and head lice haplogroups were used to construct a maximum-likelihood (ML) tree and a median-joining (MJ) network (Figs 2 and 3).

**Table 3:** Haplotype frequency of pygmies’ head lice per village in Congo-Brazzaville.

Haplotype	Pokola	Thanry-Ipendja	Béné-Gamboma	*Total*	Acc. no.
**A-5**			24	24	KX444538
**A-17**	34	30		64	KX444539
A-57	5			5	KX444540
A-58		4		4	KX444541
A-59	1			1	KX444542
**D-65**			11	11	KX444543
D-71			4	4	KX444544
D-72			1	1	KX444545
D-73			1	1	KX444546
C-74			32	32	KX444547
C-75			6	6	KX444548
C-76			3	3	KX444549
C-77			2	2	KX444550
C-78			1	1	KX444551
C-79			1	1	KX444552
***Total***	40	34	85	160	

ML and MJ analyses had similar results: all the *cytb* sequences were divided across the four major supported clades, represented by four connected subnetworks distinct groups as shown in the MJ network (Fig 2) corresponding to the known clades: A, D, B, and C. The 15 haplotypes in our study fell into all of the three haplogroups, A, D, and C.

The haplogroup A subnetwork was star-like in structure, with the most prevalent and widespread haplotype being A5 (78% of locations and 45.4% of the 1,005 analyzed human lice) in the center. 24 (15%) of our *cytb* sequences have this A5 haplotype and are all from the village of Béné-Gamboma, while a total of 64 (40%) *cytb* sequences (34 sequences from Thanry-Ipendja and 34 sequences from Pokola villages) have the A17 haplotype, which is the second most common A-haplotype and derived from the A5-haplotype by one mutation step. The remaining five clade A sequences, four from Thanry-Ipendja and five from Pokola, defined three novel haplotypes, named here A57, A58, and A59. These three novel haplotypes derived from A17-haplotype by one mutation step.

Haplogroup D, which is genetically close to A, only consists of haplotypes from Ethiopia and the Republic Democratic of Congo (RDC). The 45 (45/160) pygmy head lice sequences within clade D defined four haplotypes, of which three are novel (named here: D71, D72, D73), while the fourth haplotype possessed D65 haplotype from RDC.

The clade C, representing the most divergent lineage in which two sub-clades can be defined, here referred to as sub-clade C1, which consists of head lice from Ethiopia, France and the Asian continent, and sub-clade C2, which consists of head lice from Senegal and Mali. These two subclades are separated by 12 mutations steps. Interestingly, all 45 (45/160) pygmy head lice sequences within clade C yielded six novel haplotypes, named here as C74-C79 and are parts of sub-clade C1.

### Molecular detection of pathogens

In this study, the qPCR investigation of all 630 lice samples for *Bartonella* spp., *Rickettsia* spp., *R*. *prowazekii*, *Y*. *pestis*, and *Anaplasma* spp. produced no positive results. However, we obtained positive results when testing for the presence of *Borrelia* spp. and *Acinetobacter* spp. The DNA of *Borrelia* spp. was detected in 11/630 (1.74%) head lice collected from 7/126 (5.55%) individuals. All *Borrelia*-positive lice were clade A and found only in Pokola. The DNA of *Acinetobacter* spp. was detected in 235/630 (37.3%) head lice collected from 93/126 (73.8%) people. Of the 235 positive lice, 176 (26%) were clade A, 24 (3.8%) clade D, and 47 (7.5%) clade C. Sixty-one of these infected lice were from Pokola, forty-one from Thanry-Ipendja, and one hundred and thirty-three from Béné-Gamboma.

#### Molecular identification of *Borrelia* species

**Fig 4:**
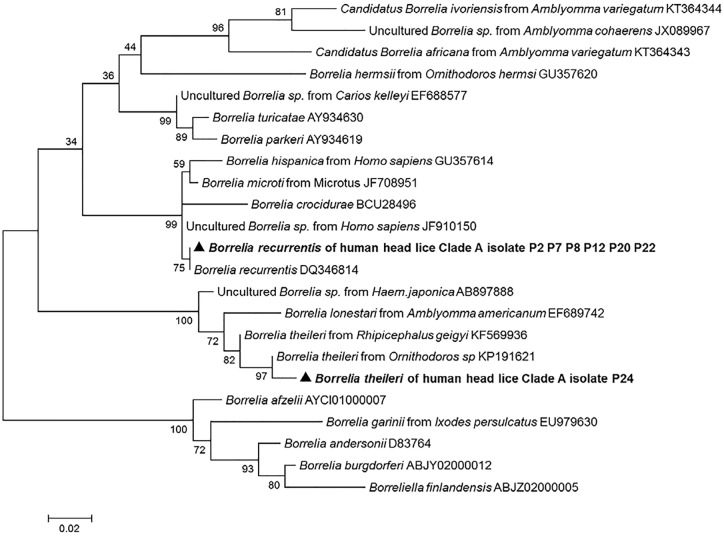
Maximum-likelihood phylogenetic tree based on 340-bp fragment *fla*B gene of the *Borrelia* species.

We succeeded in amplifying a 344-bp fragment of the *flaB* gene from all 11 lice belonging to clade A which were positive in qPCR. The comparison with the GenBank database sequences identified ten (10/11) of the obtained sequences as *B*. *recurrentis* with 100% similarity, and the one remaining sequence was identified as *B*. *theileri* with 99% identity. The phylogenetic position of these *Borrelia* is shown in Fig 4. The sequences of these two *Borrelia* were deposited in the GenBank under the accession number: KX444533- KX444534.

#### Molecular identification of *Acinetobacter* species

We succeeded in amplifying a fragment of the *rpoB* gene in 202 of the 235 samples that were positive in qPCR for *Acinetobacter* spp. The comparison of the nucleotide sequences with the GenBank database sequences revealed that only 144/202 (71.3%) sequences match eight species of *Acinetobacter* sharing 98–100% similarity, which are, in order of decreasing frequency: *Acinetobacter junii* (37/202; 18.31%), *Acinetobacter ursingii* (29/202; 14.35%), *Acinetobacter baumannii* (22/202; 10.89%), *Acinetobacter johnsonii* (19/202; 9.40%), *Acinetobacter schindleri* (17/202; 8.41%), *Acinetobacter lwoffii* (9/202; 4.45%), *Acinetobacter nosocomialis* (7/202; 3.18%), and *Acinetobacter towneri* (4/202; 1.98%). The distribution of species according clade of lice and collection site are presented in Table 4. The other 52/202 (25.74%) sequences also rated resembled *Acinetobacter* but were of poor quality, which is assumed to be due to co-infection with several *Acinetobacter* species.

**Table 4:** Detection of head lice clades and pathogens in the pygmy population in Congo-Brazzaville.

Villages	Sample no. (%)	*Acinetobacter* species		*Borrelia* species		*Moraxellaceae bacterium* Infection rate no. (%)
		Infection rate no. (%)	Species identification	Infection rate no. (%)	Species identification	
Béné-Gamboma						
Person	72 (57.1%)	53				1
Head lice	330 (52.3%)	133				2
Clade A	131	62	AJ, AU, AJn, AB, AS, AL			2
Clade D	65	24	AJ, AU, AJn, AB, AN, AS, AL			-
Clade C	134	47	AJ, AU, AJn, AB, AN, AS, AT			-
Thanry-Ipendja						
Person	18 (14.3%)	12				1
Head lice	137 (21.7%)	41				1
Clade A	137	41	AJ, AU, AJn, AB, AN			1
Pokola						
Person	36 (28.6%)	28		7		2
Head lice	163 (25.9%)	61		11		3
Clade A	163	61	AJ, AU, AJn, AB, AS, AL	11	BR (n = 10), BT (n = 1)	3
*Total*						
Person	126	93 (73.8%)		7		4
Head lice	630	235 (37.3%)		11		6
Clade A	431 (68.4%)	164 (26%)	AJ, AU, AJn, AB, AN, AS, AL	11	BR, BT	6
Clade D	65 (10.3%)	24 (3.8%)	AJ, AU, AJn, AB, AN, AS, AL	-	-	-
Clade C	134 (21.3%)	47 (7.5%)	AJ, AU, AJn, AB, AN, AS, AT	-	-	-

AJ: *Acinetobacter junii*; AU: *A*. *ursingii*; AJn: *A*.*johnsonii;* AB: *A*.*baumannii;* AN: *A*. *nosocomialis;* AS: *A*. *schandleri;* AL: *A*. *lwoffii;* AT: *A*. *towneri*.

*BR*: *Borrelia recurrentis; BT*: *B*. *theileri*.

**Fig 5:**
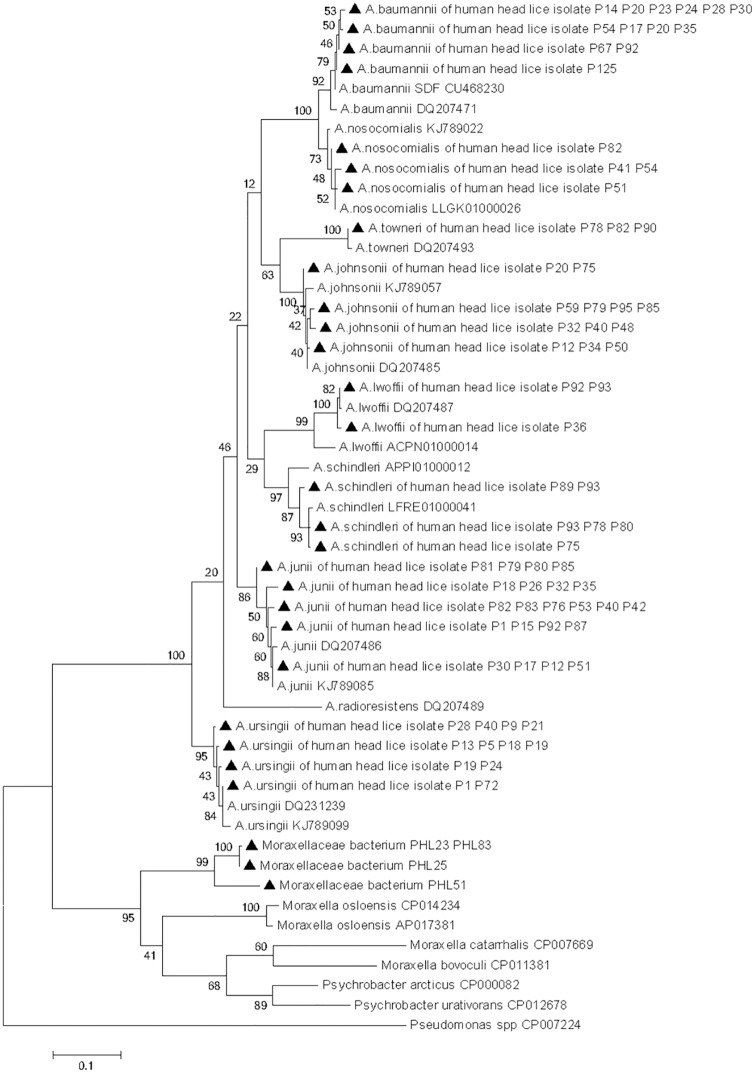
Maximum-likelihood phylogenetic tree based on 440-bp fragment *rpoB* gene of the *Acinetobacter* species and *Moraxellaceae* species, while *Pseudomonas* was used as an out group.

Six of the 202 (2.97%) remaining sequences revealed 76% identity with the sequence of *Moraxella osloensis* (accession no. AP017381). It may represent the DNA of an as yet unisolated and undescribed bacterial species of *Pseudomonadales*. The phylogenetic tree demonstrated that all *Acinetobacter* species were classified in the same group as the reference sequence strain and showed that all the *Moraxellaceae bacterium* were classified in the same group as the *Moraxella* species but formed a separate branch on the phylogenetic tree (Fig 5).

The partial *rpoB* sequences of the *Acinetobacter* species and the *Moraxellaceae* species obtained in this study were deposited in the GenBank under the accession number KX444507-KX444532 and KX444535-KX444537, respectively.

## Discussion

Here, we report the first molecular data on human head lice, *P*. *h*. *capitis*, infesting the pygmy population in the Republic of Congo in Western Africa. In this study, we established and evaluated for the first time, qPCR assay based on two duplex designed from the *cytb* gene, which is very well established in the study of lice, in order to identify all known clades of *P*. *humanus*. The assay adopted herein proved itself to be fast, specific, sensitive and fully compatible when routinely analyzing large collections of lice specimens.

The mtDNA analysis of 630 head lice, collected from 126 pygmies, showed the presence of three major mitochondrial haplogroups: A, C and D, indicating high mtDNA diversity among the head lice studied. Haplogroup A was the most prevalent (56%) followed by haplogroup C (5%). The data confirm that clade A has worldwide distribution, as reported by others [6, 8, 9, 10]. Previous studies reported that clade C is limited to Nepal and Thailand [1, 5, 23], Ethiopia, Senegal and Mali [5, 9, 18, 22]; this is the first report of clade C which has been found in the Republic of Congo. The remaining samples (10.3%) were from new haplogroup D, which is known only to exist in Democratic Republic of the Congo and Ethiopia [2, 6]. In addition to inter-haplogroup diversity, *P*. *humanus* also presents intra-haplogroup diversity, illustrated by many distinct A, B and C haplotypes [2, 12, 39]. These results are supported by our finding, that, of the 160 head lice *cytb* sequences analysed, 15 different haplotypes were identified, of which 11 were novel.

*B*. *recurrentis* is the known causative agent of relapsing fever which, if untreated, can be fatal in up to 40% of patients [13, 43, 44]. It has long been established that body lice are the main vector for this bacterial pathogen [13, 27]. In the present study, the DNA of *B*. *recurrentis* was detected in 10/630 (1.58%) head lice belonging to clade A collected from 6/126 (4.76%) individuals. Specifically, all positives cases were only found in Pokola, suggesting that a small, unnoticed outbreak may have occurred in the population in this area. This is the second report of the presence of *B*. *recurrentis* DNA in human head lice. Recently, this bacterium was also detected in 23% of head lice clade C from patients with louse-borne relapsing fever in Ethiopia and, because these patients were also infested with body lice, the authors hypothesize that head lice might be contaminated by blood that is infected with *B*. *recurrentis* [21]. In this study, the discovery of *B*. *recurrentis* in the clade A head lice, the same clade that includes body lice, and the absence of body lice may support the hypothesis that *B*. *recurrentis* may be transmitted by clade A head lice.

Nevertheless, evidence for the presence of the DNA of this bacterium in head lice by PCR cannot distinguish between transient infections, accidentally acquire the pathogen from the blood of infected individuals, and those established in a competent vector, maintain and transmit the pathogen. Further studies are needed to determine whether the head louse can act as a vector of *B*. *recurrentis*.

Interestingly, one of the *Borrelia*-positive lice was identified as *B*. *theileri*. This is the first report of the presence of the DNA of this species in human head lice. *B*. *theileri* is a spirochete that causes borreliosis in cattle, a relapsing fever-like illness, transmitted by hard ticks, such as *Rhipicephalus (Boophilus*) [45]. This infection can be considered as being rediscovered, appears to exist in regions where diagnostic ability is limited and its impact on livestock is largely unexplored [45].

In this study, two hypotheses can arise from the detection of *B*. *theileri* in human head lice. The first one is that the presence of this bacterium results from environmental and/or laboratory contaminations. This hypothesis is hardly possible, because, our work was carried out in a laboratory where *B*. *theileri* had never been worked on, nor had *B*. *theileri* DNA been extracted. Indeed, each PCR assay was systematically validated by the presence of positive and negative controls. Moreover, our collection contains lice only and didn’t contain another specimens like ticks that could be an important source of environmental contamination. The second hypothesis is that, as head lice feed only on human blood [5], the acquired infection would be from the blood of patients with ongoing bacteremia. Although, humans infected with this spirochete have not been described in the literature, the transmission of this pathogen to humans may not be ruled out. Moreover, the sequence generated in this study was more similar by *flaB* sequence comparison to those reported from *Ornithodoros* sp. soft tick (GenBank KP191621) collected from cave in Israel, than, those reported from *Rhipicephalus* hard tick (GenBank KF569936) from Mali, as shown in the phylogenetic tree (Fig 4). *Ornithodoros* ticks can feed from multiple warm-blooded vertebrates, including humans, and are known to transmit several species of *Borrelia* to humans [27, 43], thus taking in consideration that the epidemiology of *B*. *theileri* is not yet completely discovered, hypothetically it may be transmitted to humans.

Finally, if our hypothesis of *B*. *theileri* bacteremia in persons harboring head lice is true, this may merely reflect ‘accidental spill-over’ from animal hosts infection, such phenomena has already been described in the literature, with the finding of the DNA of *B*. *duttonii*, the species that is only know to infect ticks and humans, in chickens and swine living close to their human owners [43].

Findings from this study also show widespread infection of head lice with several species of *Acinetobacter*. In total, eight *Acinetobacter* species were detected in 144 samples; *A*. *junii* was the most prevalent, followed by *A*. *ursingii*, *A*. *baumannii*, *A*. *johnsonii*, *A*. *schindleri*, *A*. *lwoffii*, *A*. *nosocomialis* and *A*. *towneri*. The DNA of *A*. *towneri* was only found in clade C head lice, the DNA of *A*. *lwoffii* was only found in clades A and D, while the DNA of the remaining species was found in all three clades A, D and C.

Previous studies demonstrated that *A*. *baumannii* is the most commonly found species in body and head lice [23], as shown by its detection in 21% of body lice collected worldwide [15], in 33% of head lice collected from Parisian elementary school children, belonging to the clade A [19] and in 71% body and 47% head lice collected from healthy individuals from Ethiopia [20]. Another study, performed in head lice samples collected from elementary school children in Thailand, showed the presence of the DNA of three *Acinetobacter* species in 3.62% head lice belonging to both clade A and C. The *Acinetobacter* species identified were *A*. *baumannii*, *A*. *schindleri* and *A*. *radioresistens* [23]. When comparing the panel of *Acinetobacter* species found in all these studies with our findings, *A*. *radioresistens* was the only species that we did not identify in our head lice specimens. Conversely, our sampling showed, for the first time, the presence of the DNA of *A*. *junii*, *A*. *ursingii*, *A*. *johnsonii*, *A*. *lwoffii*, *A*. *nosocomialis* and *A*. *towneri* in human head lice, but further study is needed to determine the significance of this finding.

Furthermore, it is still unknown how these lice acquire their *Acinetobacter* infections. Some authors have argued that the infection could occur after the ingestion of infected blood meal from individuals with ongoing bacteremia, or may possibly be derived from superficial contamination through human skin while feeding [15]. An experimental study showed that the human body louse, feeding on bacteremic rabbits, is able to acquire and maintain a persistent life-long infection with *A*. *baumannii* and *A*. *lwoffii* [46]. Furthermore, another study performed a comparison between two sequenced genomes of *A*. *baumannii* and showed that the *A*. *baumannii* SDF strain, isolated from a human body louse, had several hundred insertion sequence elements which have played a crucial role in its genome reduction (gene disruptions and simple DNA loss) compared to the human multidrug-resistant *A*. *baumannii* AYE strain, and also been shown to have low catabolic capacities, suggesting the specific adaptation of this strain to the louse environment [47].

However, *Acinetobacter* species are widespread in nature (water, soil, living organisms, and the skin of patients and healthy subjects) [47], and because the frequency of with which these species associate with the skin of pygmy population is unknown, it is not possible to rule out the infection of lice by external contamination. Clinically, *A*. *baumannii* is known to be a major cause of nosocomial infections in humans and it is an increasing public health concern due to the increasing resistance to antibiotic treatment which has been identified worldwide [47]. Other *Acinetobacter* species include *A*. *lwoffii* and *A*. *junii* are also often identified as the cause of infection in humans [48]. However, it still not clear whether these *Acinetobacter* strains present in lice are the same as those that are responsible for human infections [20].

## Conclusions

In conclusion, the qPCR adopted in this study proved to be a fast, sensitive and specific tool that is fully compatible when routinely analyzing a large collections of lice specimens. Our results showed the presence of three major mitochondrial haplogroups: A, C and D, indicating high mtDNA diversity among the pygmy head lice studied. We identified the presence of a dangerous human pathogen, *B*. *recurrentis*, the causative agent of relapsing fever, in ten clade A head lice, which had not previously been reported in the Republic of Congo. Findings from this study also show the widespread infection of head lice with several species of *Acinetobacter*.

Despite several investigations into the transmissibility of numerous infectious agents, no conclusive evidence has demonstrated the transmission of disease by head lice. That said, we believe that pathogens detected in head lice may be an indirect tool for evaluating the risk of louse-borne diseases in humans.

## Supporting Information

S1 Checklist STROBE Checklist. (DOC)

S1 Table Geographic occurrences and frequencies of *cytb* haplotypes of human head and body lice. Haplotypes highlighted in blue are the newly identified haplotypes from sequences available in GenBank.
(XLSX)
