# Expression of Concern: Head Lice of Pygmies Reveal the Presence of Relapsing Fever Borreliae in the Republic of Congo

**DOI:** 10.1371/journal.pntd.0010925

**Published:** 2022-12-13

**Authors:** The PLOS Neglected Tropical Diseases Editors

This article [1] has been identified as one of a series of submissions for which we have concerns about the reported research ethics approval information and the article’s adherence to PLOS research ethics policies.

PLOS will be investigating these concerns in accordance with COPE guidance and journal policies. Meanwhile, the *PLOS Neglected Tropical Diseases* Editors issue this Expression of Concern.

## References

[1] Amanzougaghene N, Akiana J, Mongo Ndombe G, Davoust B, Nsana NS, Parra H-J, et al. (2016) Head Lice of Pygmies Reveal the Presence of Relapsing Fever Borreliae in the Republic of Congo. PLoS Negl Trop Dis 10(12): e0005142. 10.1371/journal.pntd.0005142 27911894 PMC5135033

