# Correction: Head Lice of Pygmies Reveal the Presence of Relapsing Fever Borreliae in the Republic of Congo

**DOI:** 10.1371/journal.pntd.0012561

**Published:** 2024-10-04

**Authors:** The PLOS Neglected Tropical Diseases Editors

This Correction resolves the issues underlying the Expression of Concern for the linked article [1, 2].

Following the publication of the article and Expression of Concern [1, 2], PLOS investigated concerns pertaining to the reported ethical oversight and the article’s adherence to PLOS research ethics policies.

Specifically, PLOS was concerned that the approval number 000208/MSP/CAB.15 was also reported in other publications [3,4], despite apparent differences in the aims and objectives of the studies, study locations, study populations, and types of samples collected.

Information received during follow-up discussions indicated that the study did not receive ethics review and approval, but it received permissions from authorities in the Republic of Congo. Confirming this, PLOS received copies of research authorization documents for approval numbers 00208MSP/CAB1.5 (issued by the Chief of Staff of the Congolese Minister of Health and Population) and 0094AMEFDD/CAB/DGACFAP/DTS (issued by the Director General of the Congolese Agency for Wildlife and Protected Areas). We also received a document confirming that the authors were authorized to transport samples to France. A representative from the Aix-Marseille Université commented that the Republic of Congo does not have a national research ethics committee, and stated that the Congolese institutional ethics committees that specialize in health sciences do not issue ethical opinions on research involving collection of non-invasive samples. They stated that the institutional investigation by the Aix-Marseille Université into the ethics concerns raised concluded this article meets ethical standards.

The research authorization document #0094AMEFDD/CAB/DGACFAP/DTS grants approval for access to the Lésio-Louna Nature Reserve and the Léfini Wildlife Reserve in order to collect fecal samples from great apes and lice from indigenous populations for a study into the parasitological and bacteriological interface between humans and wild animals. The research authorization document #00208MSP/CAB1.5 grants approval for a mission to the Lésio-Louna and Léfini Reserves in order to collect stool samples and lice from indigenous populations, as well as great ape excrements necessary for a study into the microbial fauna responsible for emerging diseases.

PLOS remains concerned that the study involved a vulnerable population but did not receive formal ethics review either in the Republic of Congo or in France. However, the study appears to have met Congolese requirements, and in a related case PLOS was advised that lice collection would not require Comité de Protection des Personnes (CPP) approval in France.

In light of the documentation provided, the first sentence of the ethics statement of this article is updated to: “This study did not receive ethics review or approval, but it was authorized by the Health Ministry of the Republic of Congo (000208/MSP/CAB.15 du Ministère de la Santé et de la Population, 20 August 2015) and by the Ministry of Forest Economy And Sustainable Development of Congo (0094/AMEFDD/CAB/DGACFAP-DTS, 24 August 2015).”

This Correction resolves and supersedes the prior Expression of Concern [2].
