# Supplementary material for: Head Lice of Pygmies Reveal the Presence of Relapsing Fever Borreliae in the Republic of Congo
Source: PLoS Negl Trop Dis. 2016 Dec 2;10(12):e0005142. doi: 10.1371/journal.pntd.0005142 (PMC5135033; doi:10.1371/journal.pntd.0005142)
Supplement: S1 Checklist — (DOC) [file pntd.0005142.s001.doc]

STROBE Statement—checklist

|  | Item No | Recommendation |
| --- | --- | --- |
| **Title and abstract** | 1 | (*a*) Head lice of pygmies reveal the presence of relapsing fever borreliae in the Republic of Congo |
|  |  | (*b*) An informative and balanced summary of what was done and what was found is provided in the abstract of our manuscript. *Pages 2-3* |
| Introduction | | |
| Background/rationale | 2 | Recent studies suggest that head lice, as is the case of body lice, can act as a vector for louse-borne diseases. Therefore, understanding the genetic diversity of lice worldwide is of critical importance to our understanding of the risk of louse-borne diseases |
| Objectives | 3 | Study the genetic diversity of head lice collected from African Pygmies in the Republic of Congo and to look for louse-borne pathogens in these lice |
| Methods | | |
| Study design | 4 | Head lice samples were collected from apparently healthy authochthonal individuals (pygmies) in August 2015 |
| Setting | 5 | The collections were conducted in three different villages (Thanry-Ipendja, Pokola and Béné-Gamboma) in the Republic of Congo |
| Participants | 6 | The eligibility criteria were apparently healthy pygmy individuals living in 3 villages. These involved individuals or their legal representatives in the case of children gave all necessary permits |
| Variables | 7 | The presence of *Borrelia* and *Acinetobacter* species was detected using specific quantitative real-time PCR assays, followed by standard PCR coupled to sequencing to identify species of bacteria for each positive specimen. Mitochondrial clades of lice was identified using qPCR assay developed in this study |
| Data sources/ measurement | 8* | For each variable of interest, give sources of data and details of methods of assessment (measurement). Describe comparability of assessment methods if there is more than one group— *Pages 6-9, tables 2 and 3* |
| Bias | 9 | Describe any efforts to address potential sources of bias— *Pages 15-17* |
| Study size | 10 | 630 head lice from 126 apparently healthy pygmy individuals |
| Quantitative variables | 11 | Explain how quantitative variables were handled in the analyses. If applicable, describe which groupings were chosen and why— *Pages 6-7* |
| Statistical methods | 12 | (*a*) Describe all statistical methods, including those used to control for confounding— *Not applicable* |
|  |  | (*b*) Describe any methods used to examine subgroups and interactions—N/A |
|  |  | (*c*) Explain how missing data were addressed—N/A |
|  |  | (*d*) *Cohort study*—If applicable, explain how loss to follow-up was addressed  *Case-control study*—If applicable, explain how matching of cases and controls was addressed—N/A  *Cross-sectional study*—If applicable, describe analytical methods taking account of sampling strategy—N/A |
|  |  | (*e*) Describe any sensitivity analyses—N/A |

Continued on next page

| Results | | |
| --- | --- | --- |
| Participants | 13* | We analysed 630 head lice collected from 126 pygmy individuals living in 3 villages in the Republic of Congo |
|  |  | (b) Give reasons for non-participation at each stage— N/A |
|  |  | (c) Consider use of a flow diagram— N/A |
| Descriptive data | 14* | The 126 apparently healthy pygmies were thoroughly examined for the presence of both head and body lice. All visible head lice were removed from hair using a fine-tooth comb. Lice were then collected from the clean white tissue with forceps. No body lice were found during the examination |
| Outcome data | 15* | *Cohort study*—Report numbers of outcome events or summary measures over time*— N/A* |
|  |  | *Case-control study—*Report numbers in each exposure category, or summary measures of exposure*— N/A* |
|  |  | *Cross-sectional study—*Report numbers of outcome events or summary measures |
| Main results | 16 | *Borrelia recurrentis* was detected in 10/630 (1.58%) head lice belonging to clade A collected from 6/126 (4.76%) individuals |
| Other analyses | 17 | *B. theileri* was detected in one head louse and several species of Acinetobacter were detected.  The mtDNA analysis of head lice showed the presence of 3 mitochondrial clades: A, C and D. |
| Discussion | | |
| Key results | 18 | We report the results of the first molecular screening of pygmies’ head lice in the Republic of Congo for seven pathogens and an analysis of lice mitochondrial clades. We identified the presence of a dangerous human pathogen, *B. recurrentis*, the causative agent of relapsing fever, in clade A head lice, which was not reported in the Republic of Congo. |
| Limitations | 19 | *B. recurrentis* was detected only by PCR and no attempts to culture this pathogens was made |
| Interpretation | 20 | The evidence for the presence of the DNA of *B. recurrentis* in head lice by PCR cannot distinguish between transient infections, accidentally acquire the pathogen from the blood of infected individuals, and those established in a competent vector, maintain and transmit the pathogen. |
| Generalisability | 21 | Further studies are needed to determine whether the head lice can transmit these pathogenic bacteria from person to another |
| Other information | | |
| Funding | 22 | We thank all villagers participated in the study and the personnel of the local Health Centers for their good will and IHU Méditerranée Infection for the financial support of the study. |

*Give information separately for cases and controls in case-control studies and, if applicable, for exposed and unexposed groups in cohort and cross-sectional studies.

**Note:** An Explanation and Elaboration article discusses each checklist item and gives methodological background and published examples of transparent reporting. The STROBE checklist is best used in conjunction with this article (freely available on the Web sites of PLoS Medicine at http://www.plosmedicine.org/, Annals of Internal Medicine at http://www.annals.org/, and Epidemiology at http://www.epidem.com/). Information on the STROBE Initiative is available at www.strobe-statement.org.
